# Psychological Well-Being, Cognitive Functioning, and Quality of Life in 205 Adolescent and Young Adult Childhood Cancer Survivors Compared to Healthy Peers

**DOI:** 10.3389/fpsyg.2022.860729

**Published:** 2022-05-16

**Authors:** Marta Tremolada, Livia Taverna, Sabrina Bonichini, Marta Pillon, Alessandra Biffi

**Affiliations:** ^1^Department of Development and Social Psychology, University of Padua, Padua, Italy; ^2^Department of Woman’s and Child’s Health, Pediatric Hematology, Oncology and Stem Cell Transplant Center, University of Padua, Padua, Italy; ^3^Faculty of Education, Free University of Bozen-Bolzano, Bolzano, Italy

**Keywords:** AYA cancer survivors, psychological well-being, cognitive functioning, life perceptions, healthy peers

## Abstract

The majority of the studies underlined how adolescent and young adult (AYA) Cancer Survivors had no significant differences in their well-being and quality of life compared with a control group of healthy counterparts, although French et al. (2013) found less years of education among cancer survivors. The present study aimed at comparing AYA cancer survivors and a control group of peers who had no history of serious illness, in terms of well-being, cognitive functioning, and perceptions of life. Participants in this study were 205 AYA cancer survivors, 126 males, off therapy from a mean of 10.87 years (SD = 4.91), with a mean age of 18.96 (SD = 3.08), recruited during follow-up visits and healthy counterparts (*n* = 205), matched for age and gender. They all completed self-report questionnaires: Ladder of Life, BSI-18 and Cognitive problems. Paired *t* test evidenced significant differences between survivors (Mean = 6.19; SD = 2.07) and controls (Mean = 6.88; SD = 2.02) in perceptions of quality of life regarding 5 years before the current time [*t*_(204)_ = −3.39; *p* = 0.001], with a lower level for childhood cancer survivors. Specifically, Hierarchical regression (*R*^2^ = 0.05, *p* = 0.04) identified a shorter time since the completion of treatment (β = 0.18, *p* = 0.03) and a trend of stem cell transplantation experience (β = −0.11, *p* = 0.06) as factors associated with negative perception of precedent quality of life. The AYA cancer survivors reported lower cognitive difficulties (Mean = 1.46) than controls (Mean = 1.56) [*t*_(204)_ = −3.41; *p* = 0.001]: in memory (Mean_clinical_ = 1.32 vs Mean_control_ = 1.50) [*t*_(204)_ = −4.52; *p* = 0.001], in concentration (Mean_*clinical*_ = 1.36 vs Mean_control_ = 1.54) [*t*_(204)_ = −4.66; *p* = 0.001] and in mental organization skills (Mean_*clinical*_ = 1.47 vs Mean_control_ = 1.56) [*t*_(204)_ = −2.56; *p* = 0.01], even if they had a lower educational attainment [*X*${}_{\left(9\right)}^{2}$ = 131.28; *p* = 0.001]. They showed similar satisfaction with their psychological well-being and their lives as healthy counterparts, except for past life perceptions associated with the cancer period. Important recommendations for future research and clinical suggestions could be given.

## Introduction

### Overview of Psychological and QOL Concerns Experienced by AYA Cancer Survivors

An analysis of the most recent literature presents conflicting results on studies investigating psychological well-being in adolescent and young adult (AYA) cancer survivors.

Some studies, in fact, showed that the quality of life perceived by cancer survivors was good for most of them (Elkin et al., 1997; Zeltzer et al., 2009; Tremolada et al., 2016b). In a group of studies that compared a sample of cancer survivors with a control group of healthy counterparts who had no experience of chronic disease, it was highlighted how survivors perceived themselves more positively than healthy peers. AYA cancer survivors reported fewer depressive symptoms, perceived themselves as more intelligent, and declared having more positive interpersonal relationships (Elkin et al., 1997; Teall et al., 2013). The same researchers suggested that cancer survivors were more likely to portray themselves in a more favorable light than they actually felt. Perhaps the experience of receiving invasive treatments, such as chemotherapy or surgical removal of the tumor mass, might have a positive impact on developing resilience in survivors of childhood cancer. However, also in another recent study (D’Souza et al., 2019), young adult survivors of childhood cancer and their parents did not report increased rates of anxiety or depression compared with their former classroom peers.

In fact, there are many studies that emphasized the positive consequences on psychological well-being of having childhood cancer, especially highlighting the perceived benefits and personal growth that hospitalization experience could offer during adolescence (Kállay, 2006; Tremolada et al., 2016a), also in the hematopoietic stem cell transplantation (HSCT) experience (Tremolada et al., 2018a) and how this construct could also be evaluated by adopting qualitative narrative interviews (Tremolada et al., 2018b).

A second line of studies has a more “pessimistic” view of the psychosocial well-being of these childhood cancer survivors, reporting a preponderance of negative outcomes related to the disease.

Although many surviving patients reported high levels of perceived well-being and satisfaction with their lives, in some situations there were difficulties in accepting and coping with the experience of illness. To confirm these results, the research of Zebrack et al. (2004) and his group of collaborators showed that in the group of survivors, the perception of their own quality of life was significantly lower compared to peers and they reported an increased frequency of symptoms of depression and somatic stress, particularly if they had undergone an intensive chemotherapy. The same author in 2011 found that AYA cancer survivors presented levels of physical and mental health comparable to the average of the healthy population, while their level of perceived discomfort and stress was significantly higher. People who reported feeling more uncomfortable had lower scores on the health scale and reported having more problems with symptoms of anxiety and somatization (but not depression) and a lower quality of life.

### Risk Factors of Psychological Concerns in AYA Cancer Patients

In the study of Zebrack and Landier (2011) men, compared to women, generally perceived themselves as healthier although there were no other significant gender differences, not even age at the time of the survey, where no statistically significant differences were reported.

An interesting fact, that emerged from studies on quality of life, was linked to age at the time of diagnosis: those who were older at the time of diagnosis later reported more depressive symptoms and higher levels of stress (Kazak et al., 2010; Zebrack et al., 2012), while younger (< 6 years old), female, and relapsed patients might encounter more life challenges after their disease (Sleurs et al., 2022).

Another study (Kazak et al., 2010) comparing the quality of life and health beliefs of a group of recovered adolescents (*N* = 167) with a control group of peers (*N* = 170) who had never experienced hospitalization, highlighted how survivors perceived themselves as less competent than healthy counterparts in terms of health knowledge: in particular, with respect to their perception of health, with respect to the cognitive skills related to the health topic, and the autonomy they were able to gain from parents. These results could be explained by the negative impact that anticancer treatments and prolonged hospitalization had on cognitive abilities (memory, concentration, and attention) and on ideas about the future of cancer survivors, who experienced it as more uncertain.

### Cognitive Impairment in AYA Cancer Survivors and Risk Factors

In a study on the frequency of absences and the school performance of survivors with respect to the perceived quality of life, it emerged that only the perception of one’s physical functioning (physical activity, pain, energy) was linked to worse school performance and more frequent absences from school. In fact, these patients declared that they feel more fatigued even when performing daily activities related to domestic tasks or free time (French et al., 2013). Furthermore, this study highlighted how low school performance was predictive of poor university success and difficulties in finding employment. The literature showed that young patients treated with radiation therapy associated with chemotherapy were more at risk for long-term learning difficulties and attention deficit that had a negative feedback in their orientation toward the future. Low school performance was in fact linked to lower self-esteem and worse social skills which in adulthood were reflected in difficulty in finding work, which was higher among survivors than in the general population (Crom et al., 2007). In another recent study (Meernik et al., 2020), financial hardship related to employment disruption among female AYA cancer survivors was found to be substantial.

A recent comprehensive population-based study on long-term survivors of AYA cancer (Dewar et al., 2021) evidenced a higher rate of cognitive dysfunction and psychological distress as compared to the general population. Among cancer survivors, those reporting cognitive dysfunction had greater likelihood of having psychological distress. Similarly, cancer survivors with more severe psychological distress had greater likelihood of reporting cognitive dysfunction.

### Research Gaps and Objectives

The literature highlighted how, at present, the results of studies on the quality of life of patients who survived cancer were contradictory, especially if AYA cancer survivors underwent hematopoietic stem cell transplantation (HSCT) (Reinfjell et al., 2017). It was possible to find two opposing views: a number of studies report adverse outcomes (Rourke et al., 2007; Zeltzer et al., 2008; Tai et al., 2012), others conclude that the quality of life and psychosocial adaptations were satisfactory for most of the recovered patients (Elkin et al., 1997). The conflicting results of studies on the quality of life in patients with cancer could be partially explained by the different diagnoses in the study populations, the time elapsed since diagnosis, the methods, and the diversity of tools for evaluating the dependent variables.

There is a lack of studies on the life perceptions and hopes in AYA cancer survivors and also on their cognitive functioning perceptions that could be independent from their real academic performance.

In Italy there are limited studies conducted on this topic and that adopted control groups of healthy peers. To the best of our knowledge, there is only one Italian study (Tremolada et al., 2020) aimed to understand the possible differences in well-being, cognitive, and life perceptions in adolescents in treatment for leukemia with healthy peers. This study adopted the same questionnaires and, surprisingly, found that healthy counterparts have a better perception of current life, but reported a lower hope score, more anxiety symptoms, and more perceived cognitive problems than patients. A possible protective resource could be identified in hopefulness, predictive of a good health-related quality of life (HRQoL) in the near future (Rosenberg et al., 2018). This resource was correlated with a positive sense of wellbeing and commitment to treatment, and also improved coping and self-esteem, especially in females (Cantrell and Lupinacci, 2004). The main purpose of the research is to investigate the patient’s level of well-being both on a physical-functional and psychosocial level and to identify and investigate the possible long-term consequences of the disease and the impact this may have in everyday life. Evaluation of the psychological well-being of patients supports outpatient monitoring activity related to the physical functional area, as required by the specific therapeutic protocols for each pathology.

The first group of hypotheses aims to determine if there are significant differences in well-being, life satisfaction, and cognitive functioning in AYA cancer survivors based on sociodemographic and disease parameters. We expected a lower well-being and life satisfaction in females, in older adolescents, those with fewer years off therapy, and experience of stem cell transplantation.

The second group of hypotheses aims to verify whether there are statistically significant differences between the two groups in terms of psychological well-being, life perceptions, cognitive functioning and higher educational level obtained. Based on the existing literature, we expected to find a similar trend in the two groups for psychological well-being and life perceptions. Regarding achieving higher educational qualifications and cognitive functioning perceptions, we hypothesized a worse trend for the AYA cancer survivors.

## Materials and Methods

### Participants

All eligible survivors who attended the Pediatric Hematology-Oncology Clinic of the University of Padua in the period 2015–19 were asked to participate in this project. Eligibility criteria included being treated for cancer before the age of 18, at least 5 years having passed since the last day of therapy, and being currently 15–25 years old. We excluded survivors treated for central nervous system tumors, those with learning or sensory problems or genetic syndromes, and those who were unable to complete the questionnaires independently.

The participants in the control group (*n* = 205) met the following eligibility criteria: no history of life-threatening or chronic illness or injury and absence of learning or sensory problems and other pathological aspects. The healthy peers in the control group of were enrolled in secondary schools, youth groups, and university faculties in the same region as the patients (Veneto, northeast of Italy).

### Procedure

Ethics approval was obtained from the Hospital of Padua committee considering it as an observational and spontaneous study. The day before the follow-up appointment at the Day Hospital of the Clinic, the clinical psychologist telephoned each survivor to explain the study and obtain participation consent for the next day. If the survivor was less than 18 years old, the parent was contacted before the psychologist called the participant. Upon their arrival at the clinic, survivors received a packet that included information about the study, a consent form, and a collection of questionnaires. The consent form was completed by the AYA participants, or in the case of those younger than 18 years of age, by their parents. Participants returned the questionnaires within the following 2 weeks in stamped addressed envelopes or electronically using a protected online site.

The oncologists who followed the patients and conducted the initial query in the database to identify eligible patients abstracted the necessary data from the patients’ medical records. Medical data extrapolated included: date and type of diagnosis, therapy protocol involved, age at diagnosis, hematopoietic stem cell transplantation (HSCT yes/no, type and date), date of stopping therapy, years from stop therapy, relapses (yes/no and when) and surgical intervention (yes/no and when).

### Measures

Most of the instruments used for this investigation were derived from the Childhood Cancer Survivor Study (CCSS), a cohort of 27 centers in the United States and Canada that assembled survivor samples that were sufficiently large and diverse enough to allow investigators to investigate delayed effects of treatment.

#### Ladder of Life (CCSS)

Participants had to evaluate, using a 1–10-point scale, the quality of their current life (Current life score), the quality of their life 5 years before their disease (Past life score), and how satisfied their life will be in the future (5 years after the child’s diagnosis) (Future life score). There were no cut-offs available that could be used to interpret the scores, but the scale could be easily interpreted using the following rule: an insufficient score was identified in the range 1–5; a sufficient score corresponded to 6–7; and a good score was 8–10. With this instrument, we could obtain information about individual perception of the past, the present, and the future. It had been administered to 118 Italian mothers of patients with cancer, demonstrating good global internal consistency (Cronbach’s alpha = 0.73) (Tremolada et al., 2012).

#### Brief Symptom Inventory 18

The Brief Symptom Inventory 18 (BSI-18) consisted of 18 items grouped into three dimensions of six items, serving as a screening for depression, somatization, and anxiety. Respondents were asked to comment on how they felt in the last 7 days, and each item was rated on a 5-point Likert scale from 0 (not at all) to 4 (extremely) (Derogatis, 2000). BSI-18 was used to assess psychological outcomes in long-term survivors of childhood cancer (Axia et al., 2006; Recklitis et al., 2006) and in mothers of patients under treatment for leukemia (Tremolada et al., 2013). Brief Symptom Inventory 18 ha been administered to 118 parents of patients with cancer, demonstrating good internal consistency for both the global score (Cronbach’s alpha = 0.92) and specific domains (Depression: alpha = 0.84; Somatization: alpha = 0.83; Anxiety: alpha = 0.83). Also, for this study, the psychometric parameters were good attesting at alpha values between 0.73 and 0.82.

#### Scale of Cognitive Problems

This is a 25-item questionnaire, used in another Italian study (Tremolada et al., 2013) that investigated the presence and intensity (range from 1 = “never a problem” to 3 = “often a problem”) of cognitive problems shown by AYA cancer survivors dealing with their disease experience in the last 2 weeks. The Cognitive Problems Scale has been administered to 118 Italian parents of patients with cancer, demonstrating good global internal consistency (Alpha = 0.89). A Varimax rotated confirmatory factor analysis, explaining a good proportion of the total variance (56.63%), identified five subscales. Memory (five items; alpha = 0.78); Mental disorder (eight items; alpha = 0.82); Labile mood (three items; alpha = 0.75); Impulsivity (four items, alpha = 0.73); Concentration (five items; alpha = 0.67); these five dimensions could be combined into a total score, Cognitive Problems score (25 items; alpha = 0.89). The Cognitive Problems Scale was derived from the CCSS battery, and the larger purpose of this measure was to assess the frequency of possible cognitive problems that may arise in people who were under huge stress. In this study, the alpha values showed a moderate psychometric reliability between 0.55 and 0.70.

#### Socio-Economic and Medical Data

Each participant filled out a sociodemographic questionnaire with questions about their highest year of schooling, their mother’s and father’s education, their perceived economic situation, their type of home situation, their romantic relationship, and their type of employment.

### Statistical Methods

A chi-squared test in crosstabs was used to estimate the possible sociodemographic differences between the two samples and to better understand the comparability of the samples. The two groups were matched according to gender and age, and a file was created with the matched clinic and control pairs. Descriptive measures of central tendency and variability were calculated for all relevant variables and comparisons were made between the two groups. Inferential comparisons were made between cancer survivors and control samples using a paired sample *t*-test. We ran preliminary Pearson bivariate correlations and hierarchical regression analyses to find the possible significant associations/predictions between the examined variables. Statistical significance was evaluated at the nominal level of *p* = 0.05, with adjustments for multiple comparisons, after controlling the normal distribution of the test scores.

## Results

### Characteristics of Study Cohort

Altogether, 230 of the 325 eligible survivors were informed about the study and contacted by telephone, prior to a follow-up visit. The 95 eligible survivors who the researchers did not reach in the study had changed phone number, moved from their original residential location, had no check-ups during the research period, or had no more follow-ups in the clinic. Informed consent and completed questionnaires were received from 222 individuals (response rate = 96.5%). We have no data from the eight patients who refused to participate, so any comparison between the two groups was not possible. Of these 222 survivors, 205 were matched with participants in the control group. All patients were Caucasian with a mean age of 18.96 (SD = 3.08). We matched only 205 patients with the control group because only 205 patient–healthy peer pairs with the same gender and year of birth were possible. Tables 1, 2 illustrate the sociodemographic and medical information of the participants and their families.

**TABLE 1:** AYA cancer survivors’ demographic and disease characteristics.

Characteristic		Survivors (*N* = 205)		Controls (*N* = 205)		χ ^2^; *p*
		**Frequency**	**%**	**Frequency**	**%**	
**Mean age (Years)**	**18.96 (3.08)**					
Gender	Males Females	126 79	61.5 38.5	126 79	61.5 38.5	ns
Education	0–8 years of schooling 9–13 years of schooling > 13 years of schooling	90 92 14	43.9 44.9 6.8	101 74 30	49.3 36.1 14.7	$\chi_{\left(16\right)}^{2}$ = 31.59; *p* = 0.0001
Relationship status	Engaged Single Not reported	60 95 50	29.3 46.3 24.4	81 118 6	39.5 57.6 2.9	ns
Employment (*N* = 78)	Looking for a job Part-time Full-time Not working	19 14 44 128	9.3 6.8 21.5 62.4	8 23 16 158	3.9 11.2 7.8 77.1	ns
Diagnosis	Leukemias Lymphomas (Hodgkin and non-Hodgkin) Solid tissue Other	79 65 55 6	38.5 31.7 26.8 2.9			
HSCT	No Yes	177 28	86.3 13.7			
Age at diagnosis, Mean years (SD)	7.09 (4.38)					
Years from end of therapy. Mean (SD)	10.87 (4.91)					

*Legend: Solid tissue included the following diagnoses: Hepatoblastoma, Hodgkin lymphoma, Langerhans cell histiocytosis, neuroblastoma, bone tumor, ovarian tumor, rhabdomyosarcoma, retinoblastoma, soft tissue sarcoma, Wilms tumor. HSCT, hematopoietic stem cell transplantation.*

**TABLE 2:** Demographic characteristics of the AYA cancer survivors’ families.

Characteristic		Survivors (*N* = 205)		Controls (*N* = 205)		χ ^2^; *p*
Mother’s education	5 years of schooling 8 years of schooling 13 years of schooling Degree Not reported	11 90 66 16 22	5.4 43.9 32.2 7.8 10.7	8 50 89 52 6	3.9 24.4 43.4 25.3 2.9	$\chi_{\left(9\right)}^{2}$ = 131.28; *p* = 0.01
Father’s education	5 years of schooling 8 years of schooling 13 years of schooling Degree Not reported	17 67 74 24 23	8.3 32.7 36.1 11.8 11.2	11 46 81 38 29	5.4 22.4 39.5 18.6 14.1	$\chi_{\left(16\right)}^{2}$ = 29.94; *p* = 0.02
Economic situation perceived	Low Medium High Not reported	19 85 84 17	9.3 14.5 41 8.3	25 82 97 1	12.2 40 47.03 0.5	ns
Home situation	Rent home Home ownership with mortgage Home ownership without mortgage Other Not reported	11 37 124 19 23	8.3 32.7 36.1 11.8 11.2	11 46 81 38 29	5.4 22.4 39.5 18.6 14.1	ns

### Demographic and Schooling Level Comparability Between AYA Cancer Survivors and Controls

Comparative analyses of the different areas measured by the questionnaires used in the study were carried out on the final sample made up of 205 AYA out-of-therapy and healthy peer pairs. AYAs were paired considering gender and current age: It was possible to form 79 pairs of women and 126 pairs of men, whose average age was 18.96 years (SD = 3.08).

There were differences between AYA cancer survivors and controls only in terms of educational level [${\text{X}}_{\left(9\right)}^{2}$ = 131.27; *p* = 0.0001] (Table 1). Cancer survivors had significantly less schooling years than controls. Specifically, in our data 14.7% of the participants in the control group have a bachelor’s or master’s degree, compared to 6.8% of the cancer survivors out of therapy. Furthermore, at the same age, a higher percentage of recovered young people (21.5%) claimed to be full-time workers than their peers in the control group (7.8%).

Regarding the family characteristics of the two groups (Table 2), they are quite similar to each other, except for a statistically significant difference in the level of education of the mother [X${}_{\left(16\right)}^{2}$ = 31.584; *p* = 0.01] and of the father [*X*^2^_(16)_ = 29.939; *p* = 0.02]. As can be seen from Table 2, the mothers and fathers of the AYA in the control group (68.7 and 54.1%, respectively) have a higher school diploma or degree than the mothers and fathers of the AYA in the out-of-therapy group (40.0 and 47.9%, respectively).

### The Associations of Sociodemographic and Disease Parameters With AYA Childhood Cancer Survivors’ Well-Being

There were no statistically significant gender differences compared to the other variables in the scores obtained in the BSI-18 and Ladder of Life instruments. A statistically significant difference appeared on the mood subscale [*t*_(202)_ = −2.51; *p* = 0.01; *d* = 0.43; CI 95%: −0.29/−0.12] of the Problem Scale questionnaire in which women (mean = 1.64; SD = 0.51) scored higher than men (mean = 1.47; SD = 0.47). This indicated that women have more mood-related problems, which limited them in managing daily problems.

In the control group gender differences were identified for the BSI-18 depression scale, for past Ladder of Life perceptions and for some Problem difficulties scales (Total, Disorganization, Concentration). Table 3 shows these results.

**TABLE 3:** Means and SD of depression score, past life perceptions and problem scale scores comparing males and females.

	*t*(df)	*p*	*d*	Interval confidence 95%	Gender	Mean	SD
BSI depression	−2.15 (203)	0.02	0.31	−0.41/−0.02	Females Males	0.99 0.77	0.78 0.65
Past life perception	1.72 (203)	0.04	0.25	−0.07/1.04	Females Males	6.62 7.11	1.85 2.14
Total Problem difficulties	−2.76 (203)	0.003	0.41	−0.21/0.03	Females Males	1.63 1.50	0.32 0.32
Mental disorganization	−2 (203)	0.02	0.56	−0.2/−0.001	Females Males	0.38 0.36	0.03 0.04
Difficulty concentrating	−2.34 (203)	0.01	0.5	−0.26/−0.02	Females Males	0.44 0.42	0.04 0.04

There were no significant differences on these scales according to age, while a significant positive association was found between arousal symptoms and schooling years (*r* = 0.17, *p* = 0.02).

A series of Pearson’s correlations were run to understand the possible significant associations between the scales of psychological well-being, satisfaction with life and problem difficulties and the parameters of the disease (that is, years off-therapy, age at diagnosis, HSCT yes/no of hematopoietic stem cells transplantation). Quality of life perception scores regarding 5 years before the current time were associated with age at diagnosis (*r* = −0.15; *p* = 0.02), with years off-therapy (*r* = 0.22, *p* = 0.001), and with the presence/absence of HSCT (*r* = −0.13, *p* = 0.04). A hierarchical regression model (*R*^2^ = 0.05, *p* = 0.04) identified a significance of less years off-therapy (β = 0.18, *p* = 0.03) and a trend of significance of presence of HSCT (β = −0.11, *p* = 0.06) as factors that could negatively influence the quality of life perception regarding 5 years before the current time in AYA cancer survivors.

### Psychological Well-Being and Life Satisfaction Comparability Between AYA Cancer Survivors and Controls

The third area concerned the psychological functioning and the perception of one’s current life perception, and the first hypothesis, specifically, provided that there are no differences between the two groups considered regarding the perception of one’s psychological functioning and life perception. The first analyses were carried out on the scales of the BSI-18 questionnaire and on the questions relating to the Life perception scores, aimed at investigating this type of variable. The analyses did not reveal statistically significant differences related to the psychological well-being experienced, thus confirming the starting hypothesis.

By means of the *t*-test for paired samples, a statistically significant difference emerged in the quality of Life subscale related to the 5 years before the current time [*t*_(202)_ = −3.39; *p* = 0.001] (Table 4). The recovered AYA reported to be less satisfied With their precedent quality of life (mean = 6.19; SD = 2.07) than those in the control group (mean = 6.88; SD = 2.02).

**TABLE 4:** Means and SD of life perception and problem scale scores comparing clinic and control group.

	*t* (df)	*p* value	Confidence intervals 95%	*d*	Group	*N*	Mean	SD
Current life perception	1.24 (203)	0.21	−0.11/0.51	0.15	Clinic Control	204 204	7.10 6.90	1.83 1.39
Past life perception	−3.39 (203)	0.001	−1.08/−0.28	0.40	Clinic Control	203 203	**6.19** **6.87**	2.07 2.02
Future Life perception	1.46 (198)	0.14	−0.07/0.49	0.14	Clinic Control	199 199	8.29 8.07	1.60 1.38
Total Problem difficulties	−3.41 (204)	0.001	−0.16/−0.044	0.32	Clinic Control	205 205	**1.46** **1.56**	0.29 0.33
Impulsivity	−0.78 (204)	0.43	−0.10/−0.05	0.08	Clinic Control	205 205	1.69 1.73	0.47 0.49
Labile Mood	0.013 (203)	0.99	−0.09/0.09	0.14	Clinic Control	204 204	1.53 1.53	0.49 0.48
Mental disorganization	−2.56 (203)	0.01	−0.16/−0.02	0.28	Clinic Control	204 204	**1.47** **1.56**	0.34 0.37
Difficulty concentrating	−4.66 (202)	0.0001	−0.25/−0.10	0.45	Clinic Control	203 203	**1.36** **1.54**	0.35 0.44
Memory difficulties	−4.52 (203)	0.0001	−0.26/−0.10	0.47	Clinic Control	204 204	**1.32** **1.50**	0.34 0.47

*Bold values indicate the significative mean differences comparing clinic and control group.*

The fact that no statistically significant differences emerged in the other two scales of the instrument allowed us to confirm the hypothesis mentioned above.

### Perceived Problem Functioning in AYA Cancer Survivors Compared With That in Controls

The last hypothesis, according to which patients off therapy reported greater cognitive difficulties than their peers in the control group, was investigated by taking into account the data collected with the Problem Scale questionnaire. This questionnaire evaluated the perception of one’s own general cognitive functioning and the presence of specific difficulties such as mnemonic, attentional, and mental organization.

From the analyses carried out (*t*-test for paired samples), the means that were statistically significant in comparison between the two groups are the following:

– Difficulty in solving general problems [*t*_(204)_ = −3.410; *p* = 0.001], for which the highest scores were detected for the control group (mean = 1.57; SD = 0.33) compared to the group out-of-therapy (mean = 1.46; SD = 0.30);
– Disorganization [*t*_(203)_ = −2.557; *p* = 0.01], in which the control group (mean = 1.56; SD = 0.37) scored higher than the group of Recovered young people (mean = 1.47; SD = 0.34);
– Difficulty concentrating [*t*_(202)_ = −4.666; *p* = 0.0001] for which higher scores are recorded for the control group (mean = 1.54; SD = 0.44) compared to off-therapy group (mean = 1.36; SD = 0.35);
– Memory difficulty [*t*(203) = −4.522; *p* = 0.0001] in which the non-therapy group (mean = 1.32; SD = 0.34) scored lower than the control group (mean = 1.50; SD = 0.47).

Table 4 shows these results.

## Discussion

### Brief Summary of Main Findings

The main purposes of this study were to investigate the psychological well-being, cognitive problems, and life perceptions of AYA childhood cancer survivors, and to identify the illness or sociodemographic factors associated with these outcomes.

We intended to compare the self-reported quality of life and experiences between survivors and matched controls. The main findings of this study are: (1) the lower educational attainment reported by AYA cancer survivors and by their parents comparing with healthy peers, even if recovered patients recognized less cognitive problems; (2) a good current and future quality of life perceptions in AYA cancer survivors compared with healthy peers, even if they reported a worse quality of life in the 5 years before the current time; (3) a good psychological well-being in recovered patients comparable with that of healthy peers; (4) the identification of risk factors for cognitive and psychological difficulties such as female gender, experience of HSCT, less years off therapy.

### Cognitive Functioning and Educational Attainment of AYA Cancer Survivors Compared With Healthy Peers

Cancer survivors had significantly lower educational attainment than controls. Our results were in line with the international literature, according to which a large proportion of patients out of therapy tended to terminate their studies earlier than their healthy pairs (Crom et al., 2007; French et al., 2013). The differences between cancer survivors and healthy peers in parents’ level of education could be a limit for the pairings, since it was not possible to control it, but it may also be a result of the impact of the patients’ illness on the educational and career prospects of the parents. This will be better investigated in the future with the recruitment of other control samples.

However, regarding cognitive functioning, AYA in the out-of-therapy group surprisingly perceived themselves in a more positive light, in both domains, than their peers in the control group. Higher scores corresponded to more cognitive problems. These findings did not support our original hypothesis which presumed that survivors of cancer might perceive more cognitive problems than peers. In fact, the collected data showed that they perceived themselves better than the adolescents and young adults in the control group. On the one hand, compared to healthy pairs, they declared that they have less cognitive difficulties, specifically in terms of concentration and memory, and related to mental disorganization. These data could be explained, in part, by considering the results that emerged from the comparison of the sociodemographic variables of the two groups. The out-of-therapy group had a lower educational qualification than the control group: among survivors, fewer young people had a university degree and more were already in full-time jobs. Perceiving oneself as having less difficulty in cognitive functioning could perhaps be explained by the fact that the AYA cancer survivors, whose studies had been interrupted encountered fewer cognitive problems compared to their peers in the control group, who were more committed in their education and, consequently, more concerned about this area. Another possible confirmation of what has been said so far comes from the results of the analysis of the sociodemographic variables of the families of the two groups. Parental couple of the off-therapy group had lower educational attainment than that of the control group. Probably this variable could also have influenced the choice to stop studies earlier in the young survivors, choosing to follow the parental model.

A further explanation could be due to the fact that the tools used were self-report questionnaires that detect not so much the actual cognitive abilities, but the personal perception that one has of them. It is possible that as a result of the difficult past, cancer survivors out of therapy were led to see themselves in a more positive light, sometimes overestimating their skills.

### Quality of Life Perceptions in AYA Cancer Survivors Compared With Healthy Peers

The presence of significant differences between the two groups could be expected: Life perception could be worse in the AYA cancer survivors’ group due to the possible long-term consequences of the disease, which is particularly burdensome. However, a good current life perception was shown in the clinic group despite the past traumatic experiences in childhood.

Perhaps, it was possible to advance the hypothesis that these cancer survivors continually compare their current state of health with their difficult past, so much so that they recognized a clear improvement over past years. For this reason, they tended to declare themselves satisfied with their life and cognitive functioning, even overestimating themselves. These results are in line with some studies conducted internationally (Elkin et al., 1997; Teall et al., 2013), according to which survivors were more inclined to represent themselves in a more favorable light than they really felt. Perhaps having been subjected as patients to invasive treatments, such as chemotherapy or surgical removal of the tumor mass, not only taught them to survive but also to overcome and develop in the best possible way, fighting adversity with more determination and valuing all the personal and environmental resources available to them. This phenomenon was related to the concept of post-traumatic growth, an important issue for AYA childhood cancer survivors (Tremolada et al., 2016a).

Confirming the consistency of these results, a significant difference emerged between the two groups in the area of satisfaction with the last five-years of life. Patients out of therapy declared themselves less satisfied when looking back on past experiences than healthy counterparts. These results were likely to be in line with the painful experience of illness experienced by the young people out of therapy in their past, which probably marked them compared to their peers who had never experienced with this traumatic past.

In fact, this particular group of adolescents in the past had faced challenges that left them very debilitated, and this is undoubtedly a differentiating element.

However, despite the memory of the disease still present, these AYA probably aspired to look at their present and future with positivity. Our data seemed to go more in the direction of other research (Elkin et al., 1997; Zeltzer et al., 2009; Teall et al., 2013), according to which cancer survivors, while admitting some difficulties, feel psychologically satisfied and confident for their future development.

### Psychological Well-Being in Recovered Patients Comparable With That of Healthy Peers

Regarding mental health status, in some studies a more negative profile emerged in AYA childhood cancer survivors, who declared themselves more tired in their cognitive tasks and as having self-esteem difficulties (Tremolada et al., 2017), having more difficulties at school both from the point of view of performance, with greater absences and the possibility of failing, and from the point of view of achieving higher qualifications (Crom et al., 2007; French et al., 2013; Tremolada et al., 2016b). These data could be explained by considering that the young people could not yet have gained all the resources to cope with the long-term effects that invasive therapeutic procedures had on their well-being and cognitive functioning.

However, the results obtained from the comparison between the two samples of the present research were encouraging and not in line with that part of the literature that described AYA cancer survivors as less satisfied with their lives and as having more limitations than their healthy counterparts.

From the point of view of psychological functioning, no significant differences emerged between the two groups that do not perceive themselves as different with respect to anxiety levels, physical problems, and the presence of possible depressive symptoms. A similar result was also obtained in preadolescents and adolescents during the acute therapy phase (Tremolada et al., 2020).

Both groups were equally satisfied with their current and future life perceptions: these data were comforting since it would seem that the history of illness, undoubtedly painful and difficult to process, had not left negative marks on the lives of these young people who were preparing to face their future with optimism.

### Risk Factors for Cognitive and Psychological Difficulties

Some studies highlighted the presence of gender differences among young people out of therapy in terms of perception of their health. In particular, recovered women report higher depressive and emotional symptoms (Zeltzer et al., 2009; Zebrack and Landier, 2011). Our results did partially confirm the presence of gender differences, as the literature tended to indicate. In particular, man and women did not differ in their life perceptions and well-being, but only women reported greater difficulties related to mood. On the other hand, this gender difference was more marked in the control group, where females declared more depressive symptoms, negative life perceptions and several cognitive difficulties such as difficulty concentrating and disorganization. Analyses were also conducted to evaluate differences based on age, but no significant differences emerged in any of the variables in question: even the international literature, which investigated possible differences within the off-therapy group only, did not report any age differences. To sum up possible reflections on this topic, it was possible to advance the hypothesis that the experience of lived illness in some way contributed to make this particular group of participants homogeneous regarding their way of perceiving psychosocial health and well-being both for age and also for gender.

Worse perceptions of life in the precedent 5 years were influenced by fewer years off therapy and the presence of hematopoietic stem cell transplantation. All these disease parameters could be identified as risk factors in determining negative life perception during the treatment period.

### Strengths and Limits

This study has some merits, but also some limitations.

We would like to highlight a few strengths of this study. First, we included age- and sex- matched peers as the comparative group. Both survivors and peers were asked if they had any previous hospitalization due to a serious illness before participating in the study. Second, the size and the relative homogeneity of the sample of who participated in the research were really good. Even when controlled for, the medical variables appear to have a limited association with aspects related to the psychosocial well-being of recovered patients and adolescents. Third, both groups received the same complete set of questionnaires that investigated the concept of psychological well-being and life perceptions in a multidimensional way, making it possible to develop complex health profiles.

The study has also some limitations. First, our obtained results were not always homogeneous and coherent with the literature on this topic. Probably, this non-homogeneity could be explained by the fact that, very often, in cancer studies is not usual to find a distinction between different types of cancer in pediatric age, or because uneven control groups were used (healthy pairs not matched by controlling sociodemographic variables or comparing groups such as healthy siblings, groups of patients with other pathologies, etc.) that did not always reflect the socio-demographic characteristics of AYA childhood cancer survivors. Second, the fact that the data were collected exclusively in a region of Northern Italy makes it difficult to generalize the data to the entire national context, due to social and environmental variables that could significantly affect the results collected. Third, the exclusive use of self-report questionnaires allows the rapid collection of a large quantity of information, but which risks being negatively influenced by some variables that are difficult to control, such as social desirability, with the result that collected data do not always reflect reality.

## Conclusion

Highlighting the most problematic areas could help health professionals to propose psychological and/or psychotherapeutic interventions to those patients and their families found to be most at risk and to those who explicitly request it. For example, a clinical suggestion could be to strengthen the schooling activities during the cancer treatment and in the off-therapy time to improve AYA cancer survivors’ educational attainment. It could be useful also to set up cognitive and psychological interventions especially for women, or for those that had a HSCT experience and that were nearer to the stop therapy time. The worse quality of life perceptions related to the experience of the cancer disease could be ameliorated by adopting more recreational, social and educative activities for children and adolescents during the cancer therapies.

## Data Availability Statement

The raw data supporting the conclusions of this article will be made available by the authors, without undue reservation.

## Ethics Statement

The studies involving human participants were reviewed and approved by Hospital of Padua Committee. The patients/participants provided their written informed consent to participate in this study.

## Author Contributions

MT and SB: conceptualization and methodology. MT and LT: formal analysis. MT: investigation, data curation, and writing—original draft preparation. AB: resources. LT and SB: writing—review and editing. MP and AB: visualization. SB and MP: supervision. SB: project administration. All authors have read and agreed to the published version of the manuscript.

## Conflict of Interest

The authors declare that the research was conducted in the absence of any commercial or financial relationships that could be construed as a potential conflict of interest.

## Publisher’s Note

All claims expressed in this article are solely those of the authors and do not necessarily represent those of their affiliated organizations, or those of the publisher, the editors and the reviewers. Any product that may be evaluated in this article, or claim that may be made by its manufacturer, is not guaranteed or endorsed by the publisher.
